# Robustness of brain state identification in synthetic phase-coupled neurodynamics using Hidden Markov Models

**DOI:** 10.3389/fnsys.2025.1548437

**Published:** 2025-04-24

**Authors:** Giulia Pieramico, Saeed Makkinayeri, Roberto Guidotti, Alessio Basti, Domenico Voso, Delia Lucarelli, Antea D’Andrea, Teresa L’Abbate, Gian Luca Romani, Vittorio Pizzella, Laura Marzetti

**Affiliations:** ^1^Department of Engineering and Geology, University of Chieti-Pescara, Pescara, Abruzzo, Italy; ^2^Institute for Advanced Biomedical Technologies, University of Chieti-Pescara, Chieti, Abruzzo, Italy; ^3^Department of Neuroscience, Imaging and Clinical Sciences, University of Chieti-Pescara, Chieti, Abruzzo, Italy

**Keywords:** HMM, TDE-HMM, phase coupling, brain states, simulation

## Abstract

Hidden Markov Models (HMMs) have emerged as a powerful tool for analyzing time series of neural activity. Gaussian HMMs and their time-resolved extension, Time-Delay Embedded HMMs (TDE-HMMs), have been instrumental in detecting discrete brain states in the form of temporal sequences of large-scale brain networks. To assess the performance of Gaussian HMMs and TDE-HMMs in this context, we conducted simulations that generated synthetic data representing multiple phase-coupled interactions between different cortical regions to mimic real neural data. Our study demonstrates that TDE-HMM performs better than Gaussian HMM in accurately detecting brain states from synthetic phase-coupled interaction data. Finally, for TDE-HMMs, we manipulated key parameters such as phase coupling variability, state duration, and influence of volume conduction effect to evaluate the models’ performance under varying conditions.

## 1 Introduction

Large-scale brain networks refer to patterns of synchronized activity across different brain regions (Damoiseaux et al., 2006; Menon, 2023). The dynamic nature of these connectivity patterns, often referred to as brain states, underlies various cognitive functions, from perception and attention to memory and decision-making (Loitfelder et al., 2012; Machner et al., 2021). Understanding the mechanisms that govern transitions between these states is essential for gaining insights into the neural basis of brain functions. To this purpose, data from high temporal resolution electrophysiological techniques such as magnetoencephalography (MEG) and electroencephalography (EEG) are particularly instrumental since they can capture synchronization of neural oscillations, a foundational dynamic characterizing large-scale brain networks. Specifically, phase coupling between oscillations in different brain regions is thought to play a crucial role in inter- and intra-network communication, laying the foundation of cognitive processes such as perception, attention, memory, and decision-making with their constant interplay between brain states (Fries, 2005; Lachaux et al., 1999; Varela et al., 2001). Here, we will refer to a brain state as a millisecond-scale phase coupling of neural oscillations at a specific frequency between segregated brain regions (Marzetti et al., 2019, 2023).

Once defined, such brain states can then be extracted by employing Hidden Markov Models (HMMs) (Yoon, 2009). HMMs consist of probabilistic models assuming an underlying Markov process, referring to a system transitioning across a finite number of hidden states over time. Each state generates an observable output, such as a neural signal. By modeling the temporal dependencies between these states, HMMs can be used to infer the most likely sequence of hidden states given a sequence of observations (Rabiner and Juang, 1986). One common implementation of HMMs is Gaussian HMMs, which assume that the observed data are generated from a mixture of Gaussian distributions, where each Gaussian component represents a different brain state. By estimating the parameters of these Gaussian distributions, Gaussian HMMs can identify the most likely sequence of brain states over time. Gaussian HMMs have been successfully applied to a variety of domains, including speech recognition, natural language processing, and bioinformatics. In recent years, Gaussian HMMs have also gained traction in neuroscience, where they have been used to identify different brain states from EEG, MEG, and functional magnetic resonance imaging (fMRI) data (Coquelet et al., 2022; Dang et al., 2017; Fauchon et al., 2022; Obermaier et al., 1999). However, Gaussian HMMs might not adequately capture the complex temporal dynamics of neural data. Specifically, the Gaussian HMM mainly focuses on the amplitude changes while ignoring phase coupling (Baker et al., 2014; Quinn et al., 2018). These limitations are particularly problematic for EEG and MEG studies, which rely on analyzing brain signal coupling at specific frequencies over short time windows (Marzetti et al., 2019).

To address this limitation, Time-Delay Embedded Hidden Markov Models (TDE-HMMs) have been developed (Vidaurre et al., 2018b). TDE-HMMs extend Gaussian HMMs by incorporating information from the lag version of data. This approach allows for more flexible modeling of temporal dependencies and enhances the accuracy of state inference. Furthermore, TDE-HMMs account for power covariations and phase coupling between pairs of regions (Quinn et al., 2018; Vidaurre et al., 2018b). Since phase coherence is a fundamental mechanism for cortico-cortical communication (Fries, 2005), integrating these dynamics into HMMs is expected to yield a more accurate decoding of brain states. TDE-HMMs were previously used to unveil fast transient brain states characterizing resting state activity and to characterize cortical dynamics underlying cognitive tasks, such as face recognition and working-memory processes (Baker et al., 2014; Quinn et al., 2018; Rossi et al., 2023, 2024; Seedat et al., 2020; Zhang et al., 2021) Yet, it has been shown that TDE-HMMs are primarily deriving states based on power fluctuations, rather than phase coupling-based functional connectivity (Vidaurre et al., 2018b).

Thus, a question remains regarding the extent to which TDE-HMMs can accurately detect states driven by phase-coupling based functional connectivity. While TDE-HMMs are hypothesized to provide a richer description of brain dynamics than Gaussian HMMs, no systematic comparison has been performed yet. Accordingly, the primary goal of this study is to determine whether TDE-HMMs can more accurately model ground-truth brain states, based on the phase coupling of neuronal oscillations, in a controlled synthetic environment. If so, our secondary goal is to investigate the method’s performance as the underlying dynamics become increasingly complex by manipulating phase coupling levels, phase difference variability, state duration, and volume conduction effects.

## 2 Materials and methods

### 2.1 Synthetic coupled sources generation

We simulated 78 uncoupled signals using a band-limited process. Each signal corresponded to the centroid of the i-th Automated Anatomical Labeling (AAL) (Tzourio-Mazoyer et al., 2002) cortical parcel. We generated these time series by summing sinusoids spanning the target frequency band, in our case the alpha frequency band (*F*_*c*_ = 10 ± 2 Hz). The sinusoids were produced with small frequency increments (0.01 Hz), with each sinusoid assigned an independent random amplitude and phase. A fifth-order autoregressive (AR) filter was then applied to introduce the desired temporal smoothness. The duration of simulated data for each session was five minutes, with a sampling rate of 125 Hz.

We then generated 10 distinct states, following a Markov process and based on a random transition probability matrix and a random initial probability vector (Vidaurre et al., 2018b). Concurrently, we generated a state sequence, with the duration of states at each occurrence randomly assigned from a uniform distribution over a range of interest.

Each state was characterized by a unique adjacency phase coupling matrix. This matrix outlines which cortical parcels are phase-coupled to other parcels with a specific phase difference. The number of phase-coupling connections in the adjacency matrix of the state was randomly determined from a uniform distribution. Moreover, the chosen couplings were randomly selected from all potential couplings.

For each coupling and each state, the phase difference was randomly sampled from a uniform distribution ranging from -π to π. To simulate each phase coupling within a state, we computed the analytic signals of the two sources using the Hilbert transform. During the active periods of the state, the instantaneous phase of the second source was adjusted to maintain a fixed phase difference relative to the first source, as determined by the initial random sampling. Moreover, to introduce variability to such phase difference, a random value drawn from a Gaussian distribution with zero mean and a specified standard deviation of phase difference (std-pd = 0.1, 0.3, 0.5) was added to each instance of the state occurrence. The instantaneous amplitude of the original signals was preserved to ensure that only the phase component was modified during coupling (Basti et al., 2022).

Successively, the signals were projected to the scalp using a forward model. We used the MNI template 3-shell boundary element method (BEM) head model. Then, the lead-field matrix was constructed using the positions of 126 EEG electrodes from the 10–5 international system. Source-space data was then transformed into the sensor-space by multiplying the source data with the lead-field matrix.

To emulate a realistic dataset, non-biological and biological noise were generated and combined. This was achieved by using a multivariate Gaussian distribution with zero mean and a covariance matrix:


(1) $$C_{n}=L\,L^{T}+\lambda \,I$$

where L denotes the lead-field matrix, T is the transpose operator, *I* is the identity matrix, and λ is a scalar parameter that weights the contribution of instrumental noise to the biological noise covariance matrix. The total sensor-level variance was decomposed in equation (1). The first term models the variance due to biological sources, represented by the lead-field matrix, while the second term captures the additional variance introduced by instrumental noise. We computed λ_*i*_ separately for each sensor as λ_*i*_ = 0.1 × *var* (*signal_i_*), where the subscript *i*, denotes the signal at sensor *i*, ensuring that the added noise depends on the variance of each sensor’s signal.

Later, noise was sampled from covariance matrix *C_n_*. The quality of the sensor level data was quantified by signal-to-noise (SNR) ratio and defined as follows:


(2) $$S\,N\,R=10\,l\,o\,g_{10}\,\frac{v\,a\,r\,\left(s\,e\,n\,s\,o\,r\right)}{v\,a\,r\,\left(\gamma \times n\,o\,i\,s\,e\right)}$$

where γ is the scaling factor to adjust SNR at different level. Finally, by changing γ and adding noise, signals with controlled and specific SNR levels were created.

The resulting noisy signals were then projected back to the source space. To do so, we used an 8-mm-spaced grid in the MNI space with 3,887 vertices alongside the standard BEM MNI head model to calculate the lead-field matrix. The inverse problem was solved with the array-gain beamformer approach and the ill-conditioned covariance matrix was handled with a regularization parameter which was set to 5% of the average sensor power (Westner et al., 2022). By using the AAL atlas, we reduced the dimensionality of the source voxel data to 78 cortical parcels. For each parcel, Principal Component Analysis (PCA) was applied to all voxels belonging to the parcel, and the first PC was selected to reduce the voxels time courses to the parcel signal. To reduce the effect of volume conduction, we applied leakage correction by using the innovations orthogonalization (Pascual-Marqui et al., 2017).

### 2.2 State inference

State inference was performed using two different approaches: Gaussian HMM and TDE-HMM.

In the TDE-HMM, the original data of size *P* × *T*, where *P* represents the number of parcels and *T* the time points, was expanded by incorporating lagged versions of the data. This process creates an extended matrix known as the time-delay embedded space with dimensions (*P* × (*N*_*L*_ + 1)) × (*T* − *N*_*L*_), where *N_L_* represents the number of lags. Since this increases the dimensionality, PCA was applied to reduce the data dimensionality. After PCA, the resulting time-delay embedded space has dimensions *D* × (*T* − *N*_*L*_), where *D* is the number of PCs. We retained only the PCs that represented 60% of the total variance, while the lag was set from −1 to 1 sample lag, for a total of three lags. Such a short lag length was selected to avoid excessive complexity, which would have been too computationally expensive.

In the Gaussian HMM, the association between states and observations was modeled by a zero-mean Gaussian distribution. However, prior to training and to be aligned with TDE-HMM analysis, we performed PCA to the data, and we retained only the principal components explaining 60% of the variance.

Finally, we used stochastic inference to estimate the parameters of the Gaussian HMM and TDE-HMM (Vidaurre et al., 2018a) and the most likely sequence of states was extracted using the Viterbi algorithm (Rabiner, 1989).

We ran five repetitions of the Gaussian HMM and the TDE-HMM on z-scored and concatenated sessions. The number of states prior to the inference was set to 10, since it is a prerequisite of the model learning process. After inferring the state time series, we assessed the performance of the models by correlating the ground truth data with the inferred state time series. Since the order of the states might not be the same as the ground truth across the repetitions, the states were reordered by using the Munkres’ algorithm (Munkres, 1957). These analyses were carried out using the HMM-MAR toolbox (Vidaurre et al., 2018b).

**FIGURE 1:**
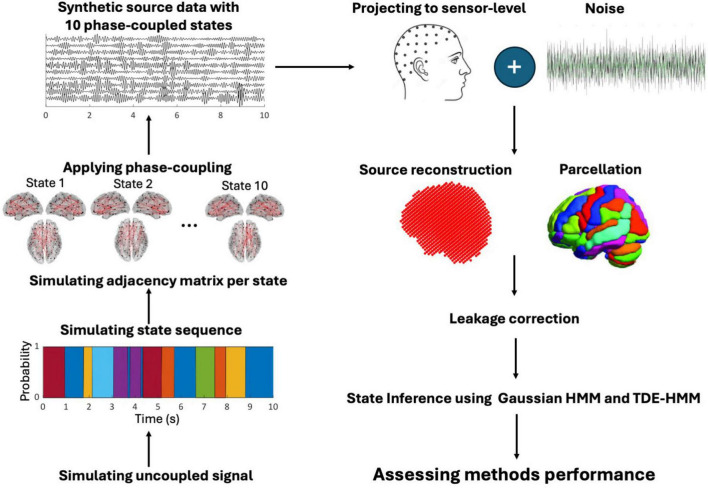
The workflow of the simulation study from left to right: we simulated the electrical activity of 78 centroid regions as uncoupled sources. Then, state sequences were defined using Markov processes, with a random duration. The adjacency matrix of a state was randomly designed based on phase-coupled connectivity. According to the adjacency matrix of a state, coupling was applied to selected sources during the occurrence of a state. Source data was projected to the sensor level adding biological and non-biological noise. The data underwent source reconstruction using LCMV beamforming reduced to parcellation. Orthogonalization was then applied to mitigate source leakage. State inference was performed using HMM and TDE-HMM. Finally, TDE-HMM performance was assessed under varying parameter settings.

Figure 1 shows the workflow of our analysis.

### 2.3 TDE-HMM performance evaluation tuning various parameters

Following a comparison between HMMs and TDE-HMMs, we explored the performance of TDE-HMMs by tuning various parameters such as SNR sensitivity (SNR levels: 3, 5, and 10 dB), phase coupling complexity (low with std-pd = 0.1, moderate with std-pd = 0.3, high with std-pd = 0.5), state duration and density, and volume conduction.

Given the superior performance of TDE-HMMs across most simulated conditions, we selected it to further evaluate the stability of HMMs at varying simulation parameters. Based on this, following the comparison stage, we examined the effect of state duration on the performance of TDE-HMMs. The simulations maintained the same SNR, phase variability levels and number of connections range as per the model comparison stage. However, two distinct ranges for state durations were tested: short (30–100 ms) and long (500–1,000 ms).

We then proceeded to change the state density, which we defined as the number of phase-coupled connections per state. Simulations were performed using two density ranges: 50–100 and 100–150 connections. State durations were kept between 30 and 1,000 ms, while SNR levels and phase variability remained consistent with the previous simulation.

Finally, we assessed the impact of volume conduction on model performance for parcellated source-reconstructed data, and orthogonalized data. Simulations were conducted by keeping the same SNR, phase variability levels, state duration and number of phase-coupled connections as at the comparison stage.

## 3 Results

Each part of this study comprised of 18 levels, based on combinations of three SNR levels, three phase variability levels, and two variations of specific parameters. These parameters included HMM type (Gaussian HMM and TDE-HMM), state duration (long and short), state density (50–100 and 100–150 phase-coupled connections), and data type (source-reconstructed and orthogonalized). To assess the differences among these 18 conditions, we performed a one-way Welch’s ANOVA. Moreover, *post-hoc* pairwise comparisons were conducted using the Games-Howell test, with the significance level set at 0.01.

### 3.1 Performance comparison of Gaussian HMM and TDE-HMM under varying SNR and phase variability conditions

**FIGURE 2:**
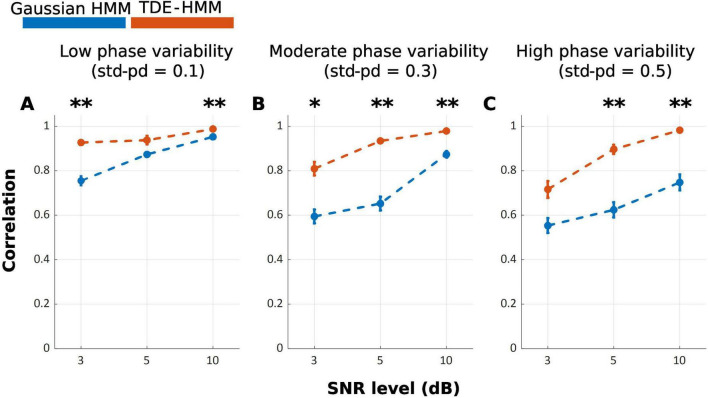
Mean correlation between inferred and ground truth state time series across repetitions, with error bars representing the standard error. **(A–C)** illustrate the effects of phase variability (std-pd: 0.1, 0.3, 0.5) at different SNR levels (3, 5, 10 dB) on the performance of Gaussian HMM (Blue) and TDE-HMM (Orange). (*p < 0.05; **p < 0.001).

Our results show that the TDE-HMM outperforms the Gaussian HMM over all conditions of phase variabilities and SNRs [*F*_*Welch*_(17.00, 316.92) = 116.98, *p* < 0.001], as shown in Figure 2. *Post-hoc* analysis indicates significant differences between Gaussian HMM and TDE-HMM over all conditions of phase variabilities and SNRs, as it is displayed in Table 1. The performance of both models improved with increasing SNR (Supplementary Table 1). Phase variability also shown an impact on performance especially at low SNR levels. Higher phase variability reduced the performance, particularly for the Gaussian HMM. In contrast, TDE-HMM was more robust to phase variability under noisy conditions (below 5 dB). Instead, at high SNR levels (5 and 10 dB), the TDE-HMM performance was relatively unaffected by high phase variability (Supplementary Table 2). Notably, under low phase variability (std-pd = 0.1) and high SNR (10 dB), the performance gap between TDE-HMM and Gaussian HMM narrowed down, indicating that both methods performed comparably well in such optimal conditions.

**TABLE 1:** Comparison of the TDE-HMM and Gaussian HMM performance over identical conditions of SNR and phase variability, based on the post-hoc Games-Howell test.

Phase variability	SNR (dB)	Mean difference	Standard error	*p*-value	0.99% confidence interval	
					**Lower bound**	**Upper bound**
0.1	3	0.172	0.023	< 0.001	0.076	0.268
	5	0.064	0.020	0.183	−0.022	0.151
	10	0.034	0.002	< 0.001	0.026	0.043
0.3	3	0.214	0.043	0.001	0.035	0.394
	5	0.281	0.031	< 0.001	0.149	0.413
	10	0.105	0.014	< 0.001	0.043	0.167
0.5	3	0.162	0.050	0.125	−0.042	0.368
	5	0.272	0.040	< 0.001	0.106	0.438
	10	0.234	0.035	< 0.001	0.083	0.386

### 3.2 Impact of state duration on TDE-HMM performance

**FIGURE 3:**
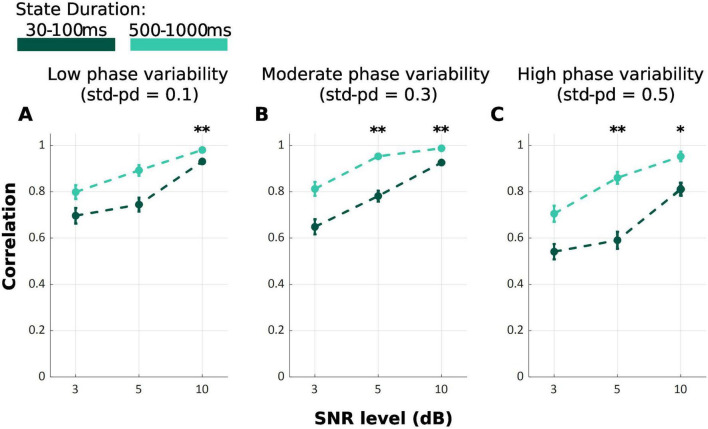
Mean correlation between ground truth and inferred state time series for TDE-HMM under varying state durations: 30–100 ms (Dark Green) and 500–1000 ms (Light Green). SNR levels are represented on the *x*-axis and phase variability levels are shown in the panels **(A–C)**. Error bars show the standard error of the correlation. (*p < 0.05; **p < 0.001).

Across all SNR and phase variability levels, TDE-HMM yielded higher performance levels at longer durations compared to shorter ones *F*_{Welch(17.00, 321.87)}_ = 129.44, *p* < 0.001 (Figure 3). However, this effect was not consistent across all conditions, as indicated by the results of pairwise comparisons using the Games-Howell test (Table 2). At SNR of 3 dB, regardless of phase variability, no significant difference is observed, exhibiting that under low SNR the capability of model decreases. In contrast, under high SNR of 10 dB, there is always significant difference.

**TABLE 2:** The difference of TDE-HMM performance, derived from pairwise comparisons using the Games-Howell, comparing long (500–1,000 ms) and short (30–100 ms) states’ duration across the three levels of SNR (3, 5, and 10 dB) and three levels of phase variability.

Phase variability	SNR (dB)	Mean difference	Standard error	*p*-value	0.99% confidence interval	
					**Lower bound**	**Upper bound**
0.1	3	0.102	0.044	0.679	−0.079	0.284
	5	0.147	0.036	0.014	−0.004	0.299
	10	0.049	0.002	< 0.001	0.041	0.058
0.3	3	0.163	0.043	0.031	−0.015	0.343
	5	0.171	0.023	< 0.001	0.073	0.269
	10	0.061	0.002	< 0.001	0.052	0.071
0.5	3	0.163	0.047	0.075	−0.031	0.358
	5	0.269	0.044	< 0.001	0.086	0.453
	10	0.141	0.033	0.006	0.004	0.278

### 3.3 Impact of state density on TDE-HMM performance

**FIGURE 4:**
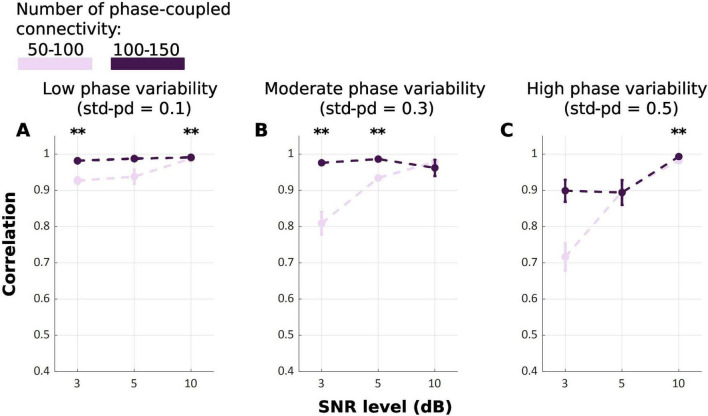
Mean correlation between ground truth and inferred state time series for TDE-HMM across repetitions for different numbers of phase-coupled connections (Light Purple: 50–100; Dark Purple: 100–150). Panel **(A–C)** refers to different phase variability levels. Error bars represent the standard error. (**p < 0.001).

Our results indicate that TDE-HMM infers states derived from 100 to 150 connections equally or even more accurately compared to the 50–100 connections condition [F_*Welch*_(17.00, 323.60) = 105.49, *p* < 0.001] (Figure 4). This indicates that increasing the number of phase-coupled connections improves the ability of TDE-HMM to accurately infer states. At high SNR level of 10 dB, the performance of TDE-HMM was high irrespective of the number of phase-coupled connections, achieving correlations close to or above 0.9 across all phase variability levels. However *post-hoc* Games-Howell test (Table 3) shows significant difference in correlations between 100–150 and 50–100 connections in 10 dB SNR at low and high phase variability. Despite these significant differences, the difference in performance is negligible. Moreover, the impact of the state density on the performance of TDE-HMM is more obvious at low SNRs, as the *post-hoc* illustrates significant difference at 3 dB SNR with low and moderate phase variabilities.

**TABLE 3:** The pairwise comparison of TDE-HMM performance between two data having various number of phase-coupled connectivity per state (50–100 and 100–150 connections) over three levels of SNR (3, 5, and 10 dB) and three levels of phase variability.

Phase variability	SNR (dB)	Mean difference	Standard error	*p*-value	0.99% confidence interval	
					**Lower bound**	**Upper bound**
0.1	3	0.054	0.009	< 0.001	0.015	0.094
	5	0.049	0.019	0.543	−0.035	0.134
	10	0.003	0.001	< 0.001	0.001	0.005
0.3	3	0.166	0.030	< 0.001	0.036	0.297
	5	0.052	0.002	< 0.001	0.039	0.064
	10	−0.016	0.022	1.000	−0.111	0.078
0.5	3	0.182	0.048	0.027	−0.014	0.380
	5	−0.002	0.040	1.000	−0.169	0.164
	10	0.011	0.001	< 0.001	0.006	0.015

### 3.4 Examining the impact of volume conduction on TDE-HMM performance across source and leakage-corrected source data

**FIGURE 5:**
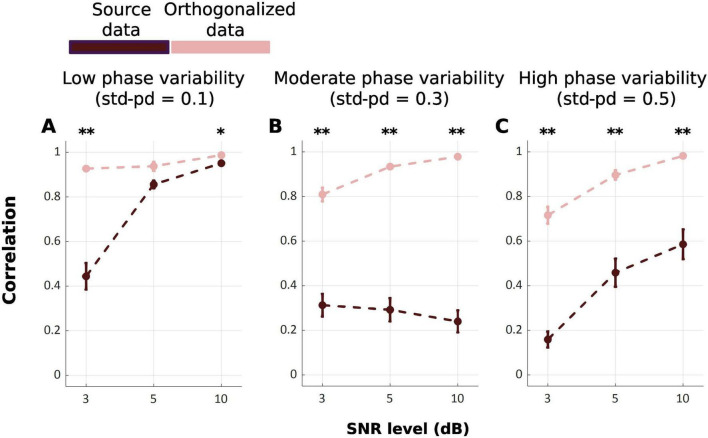
Mean correlation between ground truth and inferred state time series across source-reconstructed level (Dark Red), and orthogonalized level (Pink) under varying SNRs of 3, 5, and 10 dB and phase variability panels **(A–C)** levels (std-pd = 0.1, 0.3, 0.5). Error bars represent the standard error. (*p < 0.05; **p < 0.001).

Figure 5 displays the correlation between the ground truth state time series and the inferred state time series across repetitions for two data level: source-level, and orthogonalized-level [*F*_*Welch*_(17.00, 170.88) = 108.10, *p* < 0.001]. At the source-reconstructed level, the correlation values were consistently lower than the orthogonalized level across all SNRs and phase variability, illustrated by post-hoc Games-Howell test (Table 4). Only in one condition of low phase variability (std-pd = 0.1) and SNR = 3 dB, no significant difference was observed.

**TABLE 4:** The comparison of TDE-HMM in inferring states from signals at orthogonalized and source level data over three levels of phase variability at SNR (3, 5, and 10 dB).

Phase variability	SNR (dB)	Mean difference	Standard error	*p*-value	0.99% confidence interval	
					**Lower bound**	**Upper bound**
0.1	3	0.482	0.061	< 0.001	0.194	0.770
	5	0.081	0.027	0.216	−0.016	0.179
	10	0.036	0.004	0.001	0.016	0.056
0.3	3	0.496	0.059	< 0.001	0.234	0.758
	5	0.641	0.052	< 0.001	0.432	0.851
	10	0.738	0.049	< 0.001	0.539	0.937
0.5	3	0.557	0.051	< 0.001	0.339	0.775
	5	0.437	0.066	< 0.001	0.178	0.697
	10	0.396	0.066	< 0.001	0.130	0.662

## 4 Discussion

The primary aim of this study was to systematically compare the performance of Gaussian HMMs and TDE-HMMs in decoding functional connectivity-derived brain states from synthetic data. Our results indicate that TDE-HMMs consistently outperform Gaussian HMMs in terms of correlations between decoded brain states and ground truth. Furthermore, TDE-HMMs proved relatively resilient in non-ideal scenarios akin to those observed in real EEG data, such as lower SNR levels and high phase instability. Nevertheless, higher SNR levels tended to yield higher correlation between ground truth and estimated states, establishing the importance of SNR levels in determining the performance of both models. Phase variability further increased the uncertainty posed by low SNR, resulting in additional decreases in Gaussian and TDE-HMMs performance. Remarkably, only TDE-HMM demonstrated resilience to phase variability under high SNR level. This robustness underscores the reliability of TDE-HMM for scenarios where phase dispersion or noise could compromise connectivity detection.

One real-world scenario characterized by such unfavorable conditions is studied on clinical populations. These contexts often suffer from low SNR levels due to both pathophysiological factors and challenges in data acquisition. Nonetheless, a recent study (Rossi et al., 2024) successfully decoded clinically relevant functional connectivity states in Multiple Sclerosis patients using TDE-HMM. Given our findings of higher TDE-HMM reliability under non-ideal conditions, it is likely that the successful encoding of clinically relevant features was enabled by the method’s noise resilience.

After establishing the superior performance of TDE-HMM over Gaussian HMM, we proceeded evaluating the performance of TDE-HMM under varying conditions of noise, phase variability, state duration, and network density. We found that longer states were inferred more accurately than shorter ones, and this trend was observed across all SNR and phase variability levels. This result indicates that shorter states are more vulnerable to noise and variability, while longer states remain more stable and distinguishable. While previous studies have used HMMs to capture fast brain states (on average 50–100 ms) in resting-state data (Coquelet et al., 2022; Quinn et al., 2018; Vidaurre et al., 2018a; Vidaurre et al., 2018b), our findings suggest that decoding fast brain dynamics with TDE-HMMs should be performed only under optimal experimental conditions (i.e., low noise and phase dispersion). Nonetheless, the longer states identified here still fall within the millisecond range, enabling the exploitation of the high temporal resolution of EEG and MEG (Bai et al., 2021; Cho et al., 2024; Coquelet et al., 2022; Fauchon et al., 2022; Jiang et al., 2023).

Moreover, we established the impact of network density, as quantified by the fraction of phase-coupled connections, on the TDE-HMM performance. Specifically, we found that inferring denser states was yielding equal or even better performance compared to the lower-density states. Importantly, accurate estimation was achieved also under low SNR and high phase variability conditions. These results suggest that increasing the density of the network, and therefore its complexity, enhances the reliability of TDE-HMM in inferring states. Several studies on real MEG and High Density EEG (hd-EEG) data showed the relatively high spatial resolution of these techniques (Hedrich et al., 2017; Stoyell et al., 2021), this finding suggest that TDE-HMM analyses should be preferentially performed on such setups.

Furthermore, the impact of volume conduction on TDE-HMM performance highlighted the importance of leakage correction on source-level data. At source-reconstructed level, severe volume conduction effects resulted in poor performance, where TDE-HMM was unable to reliably infer the temporal deployment of state transitions. However, applying orthogonalization effectively mitigated leakage effects and improved the performance. Therefore, preprocessing choices could have a significant impact on TDE-HMM performance, especially the steps aimed at mitigating volume conduction.

This study has some limitations that should be addressed in future research. First, due to limited computational resources, the signals were simulated at a sampling frequency of 125 Hz. Increasing the sampling frequency could enhance the accuracy of decoding short-lasting brain states and would be particularly beneficial for investigating higher-frequency oscillations, such as gamma rhythms. Moreover, because our simulations were bound to the alpha band, future studies could examine TDE-HMM performance across other frequency ranges. In addition, due to computational constraints, we employed a TDE-HMM configuration that embedded a lag of only three samples. Extending the model’s memory by incorporating additional lag samples could further improve its ability to capture temporal dependencies and increase the accuracy of state inference. Finally, biological noise was here simulated by projecting the activity of simulated noise sources at sensor level, in the future it will be interesting to consider also noise extracted from artifactual components derived from real EEG data.

Future studies could explore using TDE-HMMs to decode latent functional connectivity brain states in real time. For instance, such states could guide brain-state-dependent, EEG-based transcranial magnetic stimulation (TMS) protocols (Marzetti et al., 2023; Vetter et al., 2023). Accurate modeling of brain states is essential for advancing state-dependent TMS, a technique that holds promise for improving outcomes in neurorehabilitation, cognitive enhancement, and psychiatric interventions (Burke et al., 2019; Rossi et al., 2009).

## 5 Conclusion

This study highlights the superior performance of TDE-HMMs compared to Gaussian HMMs at inferring hidden brain network across both favorable and unfavorable scenarios involving SNR, state duration, and phase variability. In general, TDE-HMMs exhibited relative resilience to unfavorable conditions, with performance drops becoming evident only under worst-case scenarios. Moreover, TDE-HMMs performed consistently better under high network density ground-truth conditions and when using orthogonalized source data instead of sensor data, providing insights on optimal methodological choices for their effective implementation. Overall, this study validates the robustness of TDE-HMM for inferring state time series representing phase-coupled brain networks and demonstrates the foundation for its application on real-world EEG data.

## Data Availability

Datasets will be made available by the authors upon request. The synthetic data supporting the conclusions of this article will be made available by the authors, without undue reservation.
