# Supplementary material for: Robustness of brain state identification in synthetic phase-coupled neurodynamics using Hidden Markov Models
Source: Front Syst Neurosci. 2025 Apr 24;19:1548437. doi: 10.3389/fnsys.2025.1548437 (PMC12058723; doi:10.3389/fnsys.2025.1548437)
Supplement: Supplementary file 1 [file Table_1.docx]

Supplementary Material

Table S1. The result of post-hoc analysis based on the Games-Howell test, assessing the impact of SNR variability on performance of TDE-HMM.

| Phase variability | SNR | SNR | Mean difference | Standard Error | *p* value | 0.95% Confidence Interval | |
| --- | --- | --- | --- | --- | --- | --- | --- |
|  |  |  |  |  |  | Lower Bound | Upper bound |
| 0.1 | 10 | 3 | 0.06 | 0.01 | **<0.001** | 0.03 | 0.09 |
|  |  | 5 | 0.05 | 0.02 | 0.544 | -0.02 | 0.12 |
|  | 5 | 3 | 0.01 | 0.02 | 1.000 | -0.07 | 0.09 |
| 0.3 | 10 | 3 | 0.17 | 0.03 | **<.001** | 0.06 | 0.28 |
|  |  | 5 | 0.04 | 0.01 | **<.001** | 0.03 | 0.05 |
|  | 5 | 3 | 0.12 | 0.03 | 0.017 | 0.01 | 0.24 |
| 0.5 | 10 | 3 | 0.27 | 0.04 | **<0.001** | 0.13 | 0.40 |
|  |  | 5 | 0.08 | 0.02 | 0.018 | 0.01 | 0.16 |
|  | 5 | 3 | 0.18 | 0.04 | **0.008** | 0.03 | 0.34 |

Table S2. The post-hoc Games-Howell test use to evaluated the effect of phase variability on performance of TDE-HMM.

| SNR | Phase variability | Phase variability | Mean difference | Standard Error | *p value* | *0.95%* Confidence Interval | |
| --- | --- | --- | --- | --- | --- | --- | --- |
|  |  |  |  |  |  | Lower Bound | Upper bound |
| 3 | 0.1 | 0.3 | 0.12 | 0.03 | 0.046 | 0.00 | 0.23 |
|  |  | 0.5 | **0.21** | **0.04** | **<0.001** | **0.07** | **0.35** |
|  | 0.3 | 0.5 | 0.09 | 0.05 | 0.894 | -0.08 | 0.26 |
| 5 | 0.1 | 0.3 | 0.01 | 0.02 | 1.000 | -0.07 | 0.08 |
|  |  | 0.5 | 0.04 | 0.03 | 0.994 | -0.06 | 0.14 |
|  | 0.3 | 0.5 | 0.04 | 0.02 | 0.941 | -0.04 | 0.16 |
| 10 | 0.1 | 0.3 | **0.01** | **0.001** | **<0.001** | **0.01** | **0.012** |
|  |  | 0.5 | **0.01** | **0.001** | **0.003** | **0.001** | **0.01** |
|  | 0.3 | 0.5 | -0.001 | 0.001 | 0.587 | -0.01 | 0.002 |
